# Motivating the unmotivated: how can health behavior be changed in those unwilling to change?

**DOI:** 10.3389/fpsyg.2015.00835

**Published:** 2015-06-16

**Authors:** Sarah J. Hardcastle, Jennie Hancox, Anne Hattar, Chloe Maxwell-Smith, Cecilie Thøgersen-Ntoumani, Martin S. Hagger

**Affiliations:** Health Psychology and Behavioural Medicine Research Group, Faculty of Health Sciences, School of Psychology and Speech Pathology, Curtin UniversityPerth, WA, Australia

**Keywords:** health psychology, behavioral medicine, behavior-change interventions, amotivation, motivational interviewing

Many individuals do not engage in health-promoting behaviors that would confer important health benefits despite research that has shown that engaging in a suite of four health behaviors (physical activity, eating a healthy diet, not smoking, drinking alcohol in moderation) leads to a 11–14 year delay in all-cause mortality (Khaw et al., 2008; Ford et al., 2011). Motivating people disinclined to engage in health behavior presents a significant challenge to public health practitioners. Although there have been advances in interventions to increase individuals' motivation to engage in health-related behaviors, gaps in knowledge exist. In particular, effective strategies to promote behavior change in individuals with little or no motivation to change are relatively scarce.

Most social psychological theories applied to health behavior change tend to assume a degree of motivation for change and have focused on attempts to promote action by converting motivation into action. Approaches such as goal-setting (Locke, 1996; Fenner et al., 2013), self-monitoring (Miller and Thayer, 1988), action planning (Schwarzer, 2014), and implementation intentions (Gollwitzer, 1999; Hagger and Luszczynska, 2014) focus on harnessing motivation and promoting action in those already likely to be motivated to change. As a consequence, such approaches are heavily dependent on individuals having some motivation to change even though they are not actually engaging in the behavior. These individuals are best characterized as “inclined abstainers” (Orbell and Sheeran, 1998) or “unsuccessful intenders” (Rhodes and de Bruijn, 2013). The approaches, however, do not focus on individuals with low or no motivation to change which account for a substantive proportion of the population. For example, less than 10% of smokers report wanting to quit (Wewers et al., 2003) and 60% of smokers do not make a quit attempt during any given year (Centers for Disease Control and Prevention, 2007). Similarly, up to 30% of individuals express no intention to exercise (Ronda et al., 2001; Rhodes and de Bruijn, 2013). It is clear, therefore, that a large number of individuals are not motivated to engage in health-promoting behaviors and tend to be those most at risk. In this article, we briefly review theoretical perspectives focusing on individuals who are not motivated to engage in health-promoting behaviors. We contend that although theories identify low motivation as a state, they do not provide complete explanations of, and underlying reasons for, the absence of motivation, nor do they suggest comprehensive strategies that may engage these hard-to-reach individuals. We offer some theory-derived suggestions on how to engage unmotivated individuals to increase their participation in health-promoting behaviors.

Two prominent theoretical perspectives offer conceptualizations of “unmotivated” individuals: self-determination theory and the transtheoretical model. Self-determination theory (Deci and Ryan, 1985, 2000) distinguishes between different types of motivation or reasons underlying behavioral engagement (Chatzisarantis et al., 2007, 2008). According to the theory, the state in which an individual lacks intention to act is termed *amotivation* (Vallerand, 2001). Individuals reporting being amotivated toward health behaviors are unable to identify the reasons why they act, and tend to have low intentions and poor uptake and adherence to health behaviors (Thøgersen-Ntoumani and Ntoumanis, 2006). Similarly, the transtheoretical model identifies several stages that characterize individuals on a continuum of change with respect to health behavior (Prochaska et al., 2005). Individuals in the precontemplation stage have no apparent interest in engaging in health behavior. Individuals in this stage do not consider the need of change and are resistant to suggestions of change. Some theorists have drawn parallels between the precontemplation stage and amotivated states (Thøgersen-Ntoumani and Ntoumanis, 2006). Both perspectives do not provide explicit solutions to addressing individuals in amotivated and precontemplative states. For example, interventions based on the transtheoretical model for precontemplators have tended to be limited to targeting the experiential processes of consciousness raising and dramatic relief that amounts to the information provision, both of which have limited effectiveness in changing behavior in those with low motivation (Foster et al., 2005; Miller and Rollnick, 2013; Peters et al., 2013). We argue that improving intervention effectiveness for unmotivated individuals should begin with an analysis of the underlying reasons for being in an amotivated or precontemplative state when it comes to health behaviors and how these may be specifically targeted in interventions.

Some research has examined the etiology of amotivation from a self-determination theory and social-cognitive perspectives (Pelletier et al., 1999; Vlachopoulous and Gigoudi, 2008; Shen et al., 2010). Amotivation may stem from low levels of self-efficacy, outcome expectancies, effort beliefs, and value beliefs (Vlachopoulous and Gigoudi, 2008; Shen et al., 2010). Low self-efficacy relates to low confidence and feelings that the individual lacks the capacity or resources to produce the desired behavior. Low outcome expectancies relate to beliefs that the costs of the behavior outweigh the benefits. A lack of effort beliefs is concerned with the recognition of the required amount of effort or energy needed to change behavior (e.g., perceiving physical activity as “too hard”), or overcome the perceived barriers and disinhibiting factors (e.g., fear of embarrassment, lack of knowledge), and being willing to invest the necessary effort to achieve the desired outcome. Further, low value beliefs relate to not attaching sufficient value to the behavior to make it worthwhile pursuing (Wigfield and Eccles, 2000). Low outcome expectancies and value beliefs, therefore, serve as demotivating factors. These sets of beliefs provide clear direction regarding the conditions that lead to the development of amotivation and how they could be addressed. Based on these findings, strategies aiming to reduce amotivation could include confidence-building strategies, targeting decisional balance and also those that focus on changing effort and value beliefs. Given that these types of strategies have been used in counseling approaches to changing behavior, such as motivational interviewing (Miller and Rollnick, 2013), it raises the possibility that these may be viable avenues to resolve unmotivated states like amotivation.

Motivational interviewing (Miller and Rollnick, 2013) is a counseling approach to behavior change. It is well suited to those unmotivated to change as it focused on building motivation for, and reducing resistance to, behavior change (Hardcastle et al., 2008, 2013). The interpersonal style and behavior of the practitioner are central to motivational interviewing (Hagger and Hardcastle, 2014). Few approaches are explicit about the importance and impact of the relational style in which interventions are delivered, particularly for those who are not motivated to engage in health behavior. The specific relational motivational interviewing techniques that may be useful when working with those less motivated to change include: reframing, overshooting, coming alongside, shifting focus, and emphasizing autonomy. The content-related techniques that could be adopted are those that seek to elicit “change talk” (arguments for change) and reduce “sustain talk” (the person's own arguments for not changing). These techniques include “running head start,” “looking forward,” and “values exploration.” “Running head start” is used to elicit client motivational talk through the counselor first asking open questions to explore the pros of the status quo, in order to then query the cons of the status quo. The client is also asked about the cons of changing followed by the pros of changing their behavior. “Looking forward” is a strategy to build motivation by the counselor prompting the client to envision two possible futures and deemed to be very useful in a physical activity intervention (Hardcastle et al., 2012). The first future is if they continue on the same path without any changes. The second future is if they decide to make a change and prompting them to consider “what that future may be like: if you did decide that now is not the time to change and we meet up in five years from now, what would things be like for you? What about that concerns you the most?” And “If you were to change, what would life be like in the future? How would you feel? How would things be different?” “Values exploration” is a strategy for evoking motivation by having clients describe their most important life goals and values (Miller and Rollnick, 2013). Example questions include: what things are most important to you?” or “what do you want most in life?” and “how does your (behavior) fit in with your goals and values?” Focusing on discrepancies between ideal life conditions and actual conditions may induce a desire to “recalibrate” daily behaviors to be more congruent with deeply-held, amotivated beliefs. Focusing on ideals can help decrease clients' defensiveness and foster motivation for change by transferring the focus away from “bad” behaviors or lifestyle, toward a focus on a more deeply satisfying lifestyle that can be pursued and enjoyed. The values exploration technique does not appear to have been adopted in motivational interviewing interventions outside of the substance abuse field and only one study was located that specifically explored the effectiveness of a values exploration technique in a weight loss intervention (e.g., Webber et al., 2008).

Other perspectives on changing behavior in individuals that are unmotivated to engage in health behaviors come from dual-process theories of action. According to this perspective, behavior is driven by two processes: conscious consideration of the pros and cons or expectancies of the value of engaging in the behavior relative to potential costs of doing so, and non-conscious processes that are spontaneous, impulsive, and occur with little deliberative thought (e.g., Strack and Deutsch, 2004; Hagger and Chatzisarantis, 2014, 2015 Hagger et al., 2015). Many unhealthy behaviors including unhealthy eating, smoking, and drinking excess alcohol have been conditioned by cues in the environment paired with the concomitant reward-based outcome, usually determined by dopaminergic pathways in the brain which serve as powerful reinforcers of the cued-up behavior (Rebar et al., 2015). As such, exerting conscious control to override these strong neural relations between cue and action is difficult and requires considerable cognitive resources and motivation (Hagger, 2010, 2014; Loftus et al., 2015; Rebar et al., 2015). By implication, low resources and motivation to engage in conscious effort to resist the powerful cue-driven urges makes behavior change extremely difficult. This means that individuals that are unmotivated to engage in health-related behaviors are unlikely to change because they are not motivated to invest effort in overriding the highly-automated non-conscious cue-driven processes that drive their behavior. In such circumstances, researchers have indicated that it may be important to structure individuals' environments so as to make engaging in the undesired behavior much more difficult (Sallis et al., 2012). Examples of environmental solutions that may change behavior among the unmotivated without engaging in costly persuasive techniques include: bans on smoking in public places and the workplace, employers locating car parks a distance from workplaces so employees walk a given distance to work each day, and limiting the number of alcoholic beverages that can be served in bars. Such legislation requires considerable will among policymakers and is not necessarily a universal solution. For example, banning smoking in public places and workplaces is unlikely to affect smoking at home. Environmental strategies may form part of a comprehensive package of solutions to changing behavior in the unmotivated.

To conclude, we contend that current theoretical perspectives on behavior change do elaborate sufficiently on how to approach individuals with low motivation to participate in health behavior. We have proposed some possible suggestions for future research on how to potentially engage individuals who are unmotivated to participate in health-promoting behaviors. These strategies outlined include the targeting of self-efficacy, outcome expectancies, effort and value beliefs; motivational interviewing techniques including strategies like running head start, looking forward, and values exploration, and we recommend their use in health behavior interventions that target those unmotivated to change. We also recognize that environmental interventions have a crucial role to play in promoting health behavior change among the unmotivated. It is important to note that these strategies may assist in increasing motivation among individuals to initiate a health related behavior. Increasing motivation is an important first step among amotivated or precontemplative individuals who do not engage in any health behaviors. Further strategies, however, may be needed to assist in the enactment of the behavior (e.g., planning, volitional strategies) and maintain it over the longer term (e.g., self-monitoring and self-reinforcement).

## Author contributions

SH took the lead role in conceiving and developing the ideas presented in the article and drafting the article; MH assisted in the conception of the article and took a lead role in editing and drafting the article; JH, AH, CM, and CT assisted in providing ideas for the article and assisted in drafting the article.

### Conflict of interest statement

The authors declare that the research was conducted in the absence of any commercial or financial relationships that could be construed as a potential conflict of interest.
